# Talocalcaneal Ligament Reconstruction Kinematic Simulation for Progressive Collapsing Foot Deformity

**DOI:** 10.1177/10711007231213361

**Published:** 2023-12-11

**Authors:** Sylvano Mania, Silvan Beeler, Stephan Wirth, Arnd Viehöfer

**Affiliations:** 1 Department of Orthopaedics, Balgrist University Hospital, University of Zürich, Zürich, Switzerland

**Keywords:** Biomechanics, kinematic simulation, subtalar joint, progressive collapsing foot deformity

## Abstract

**Background:**

In progressive collapsing foot deformity (PCFD), an internal and plantar rotation of the talus relative to the calcaneus may result in painful peritalar subluxation. Medial soft tissue procedures (eg, spring ligament repair) aim to correct the talar position via the navicular bone if bony correction alone is not sufficient. The effect of the medial soft tissue reconstruction on the talar reposition remains unclear. We hypothesized that a subtalar talocalcaneal ligament reconstruction might be favorable in PCFD to correct talar internal malposition directly. This pilot study aims to evaluate the anatomical feasibility and kinematic behavior of a subtalar ligament reconstruction in PCFD.

**Methods:**

Three-dimensional surface model from 10 healthy ankles were produced. A total of 1089 different potential ligament courses were evaluated in a standardized manner. A motion of inversion/eversion and talar internal/external in relation to the calcaneus were simulated and the ligament strain, expressed as a positive length variation, for each ligament was analyzed. The optimal combination for the ligament reconstruction with increased length in internal rotation of the talus, isometric kinematic behavior in inversion/eversion, and extraarticular insertion on talus and calcaneus was selected.

**Results:**

A laterodistal orientation of the talar insertion point in respect to the subtalar joint axis and laterodistal deviation of the calcaneal insertion point presents the highest ligament lengthening in internal talar rotation (+0.56 mm [3.8% of total length]) and presented a near-isometric performance in inversion/eversion (+0.01 to −0.01 mm [0.1% of total length]).

**Conclusion:**

This kinematic model shows that a ligament reconstruction in the subtalar space presents a pattern of length variation that may stabilize the internal talar rotation without impeding the physiological subtalar motion.

**Clinical Relevance:**

This study investigates the optimal location, feasibility, and kinematic behavior of a ligament reconstruction that could help stabilize peritalar subluxation in progressive collapsing foot deformity.

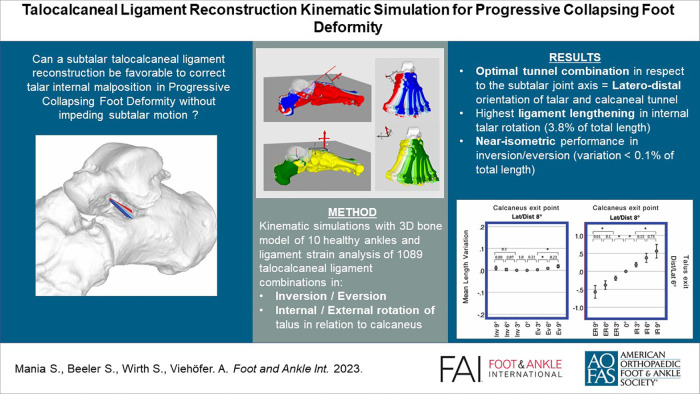

## Background

Progressive collapsing foot deformity (PCFD), or adult acquired flatfoot deformity, is characterized by a valgus alignment of the hindfoot and loss of the medial longitudinal arch.

Recent weightbearing computed tomography (CT) analyses provided more insights in the complex 3-dimensional (3D) deformity occurring in PCFD. The subluxation of the peritalar bones is characterized by a reduced joint coverage in the anteromedial and posterior talar facet, as well as in the calcaneocuboid and talonavicular joint.^2,15,16^ As such, a quantification of the joint coverage of middle facet of the subtalar joint can be a reliable diagnostic marker for peritalar subluxation.^7^ It has been demonstrated that orientation of this deformity is characterized by a medial shift, or internal rotation, of the talus relative to the calcaneus, which leads to an impingement in the sinus tarsi.^15,16^

Several surgical procedures, including lateral column lengthening and medializing calcaneal osteotomy, aim to correct this subluxation with bony correction and constitute the current gold standard in treatment of PCFD. Despite the lack of large long-term follow-up studies, a spring ligament reconstruction has been recommended as a soft tissue procedure to realign the talonavicular joint and has been shown to be the only procedure able to correct the foot ankle offset during weightbearing-CT analysis.^4,6,8,32^ Noticeably, such a reconstruction only acts as a hammock sustaining the talar head and pushing it back onto the calcaneus without directly connecting the talus with the calcaneus.

Several case reports or technical notes have described a reconstruction of the interosseous talocalcaneal ligament (ITCL) to stabilize the subtalar joint and a prospective study with a nearly similar concept proposed a reconstruction of the cervical ligament (CL) reconstruction.^10,12,18,23,30^ However, both these surgical techniques and the current literature are focused on the treatment of subtalar instability in the setting of lateral ankle instability and not medial-sided pathology such as PCFD.^21^

Four main ligaments may play a role in subtalar instability ITCL, CL, anterior capsular ligament (ACaL) and calcaneofibular ligament (CFL), which all concurrently play a role in the stability of the subtalar joint.^22^ The ACaL and ITCL, which are near and often confused, work as a complex and maintain the apposition between the 2 bones.^5,31^ The fibers of the CL are tensed during hindfoot inversion and with anterior drawer in neutral position, and its location in the sinus tarsi are far away from the subtalar rotation axis, which is a reason why some authors have attributed an important stabilizing role to it.^12,17,20,23^ The CFL, which is outside from the sinus tarsi, stabilizes the subtalar and the tibiotalar joint because of a tension in inversion and dorsiflexion.^14^ A recent study highlighted the presence of CL and ITCL lesions in 60.3% and 44.9% of PCFD cases, respectively, underlining a potentially underestimated role of the subtalar ligaments in PCFD.^13^

## Purpose or Hypothesis

This pilot study aims to evaluate the anatomical feasibility and kinematic behavior of a subtalar ligament reconstruction in PCFD. It investigates the optimal placement of a ligament reconstruction, showing minimal ligament strain during inversion/eversion while having maximal strain during a subtalar subluxation with internal rotation of the talus. Additionally, the study examines ligament strain during subluxation with external rotation of the talus.

We hypothesize that a subtalar talocalcaneal ligament reconstruction might be favorable to correct talar internal malposition directly in PCFD without impeding the physiological subtalar motion.

## Material and Method

This study has been approved and registered by our local ethic commission (BASEC Number 2022-01474) and is in accordance with the ethical standards of the 1964 Declaration of Helsinki.

The ligament reconstruction was simulated in 3-dimensional surface models from 10 healthy ankles. Different ligament insertional points on the calcaneus and talus were simulated using the intersection point of a stepwise rotated subtalar axis and the bone. To analyze the strain of these ligament reconstructions in physiological inversion/eversion and pathologic subluxation (external rotation in respect to the calcaneus), the length variation of the ligaments was calculated throughout these movements.

The optimal combination for the ligament reconstruction with increased length in internal rotation of the talus, isometric kinematic behavior in inversion/eversion, and extraarticular insertion on the talus and calcaneus was finally selected.

The exact method of model segmentation, movement and ligament simulation, and statistical analysis are described below.

### Model Segmentation

3D surface models of entire feet were segmented from CT scans (Siemens SOMATOM Definition AS, Siemens Healthcare, Erlangen, Germany) of 10 patients with healthy ankles. The CT scans were retrospectively selected from a database of patients requiring patient-specific cutting guides based on a healthy foot for various indications of foot osteotomy on the opposite side (leg, ankle, and hind-/forefoot osteotomy). None of these patients report pain, bone injury, or previous surgery of the foot, and no cavovarus or flat foot deformity was clinically observed. Six of the 10 patients were male, with an equal distribution of right and left foot, and the mean age was 48.6 years (SD 6.4). The segmentation was generated with a commercial segmentation software (Mimics 19.0; Materialise NV, Leuven, Belgium) using thresholding, region growing, and the marching cubes algorithm. The produced 3D models were imported into an in-house-developed planning software (Balgrist CARD AG, Zürich, Switzerland), allowing an unrestricted navigation and placement of reference points on the bone surface. The cartesian coordinate of the placed reference points were recorded to perform various computations.

### Movement simulation

**Figure 1.**
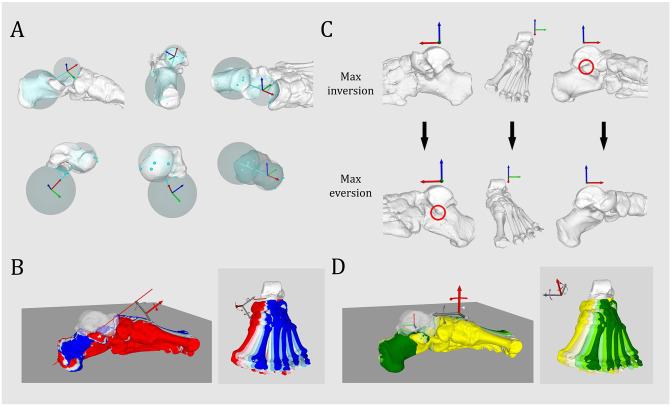
Simulation of peritalar rotation. (A) Simulation of subtalar rotation axis based on the talar facet of the calcaneus and the talar facet of the navicular bone (from Mania et al,^19^ no permission needed). (B) Simulation of progressive inversion/eversion along the subtalar rotation axis, respectively, from red to blue at 9, 6, and 3 degrees inversion, neutral position, and 3, 6, and 9 degrees eversion. (C) Bone impingement (red circle) occurring between the lateral talar process and the anterior process of the calcaneus in maximal eversion (24 degrees eversion), bone impingement occurring between the posteromedial talar process and the sustentaculum tali in maximal inversion (18 degrees inversion). (D) Simulation of peritalar internal/external rotation along a vertical axis located at the talocrural rotation axis, respectively, from dark green to yellow, 9, 6, and 3 degrees internal rotation, neutral position, and 3, 6, and 9 degrees external rotation. [See online article for color figure.]

The subtalar rotation axis was defined as a line joining 2 spheres fitting the talar surface of the calcaneus and the talar surface of the navicular bone with the help of reference points standardly placed on the bone surface, as described by Mania et al^19^ following a modified method of Parr et al^26^ (Figure 1A). This axis was used to simulate an inversion of 9 degrees with incremental change of 3 degrees until an eversion of 9 degrees (Figure 1B). The total range of motion (ROM) of the subtalar joint in inversion-eversion has been reported to be 13.9 ± 3.5 degrees according to in vivo data with 3D weightbearing CT, 15.1 ± 9.7 degrees, or even 37.3 ± 5.9 degrees.^3,24,29^ We arbitrarily choose a lower ROM of 18 degrees for all ankles to avoid a bone impingement between the posteromedial talar process and the sustentaculum tali in maximal inversion, or between the lateral talar process and the anterior calcaneal process in maximal eversion (Figure 1C).

A vertical rotational axis along the tibia was used to simulate the subluxation of the talus in relation to the calcaneus. An internal talar rotation of 9 degrees with incremental change of 3 degrees until an external rotation of 9 degrees was simulated (Figure 1D). The ROM was limited to 18 degrees to avoid a bone impingement between the medial talar facet and the sustentaculum tali in maximal talar internal rotation, and between the lateral talar process and the posterior calcaneal surface in maximal talar external rotation.

### Ligament Simulation

**Figure 2.**
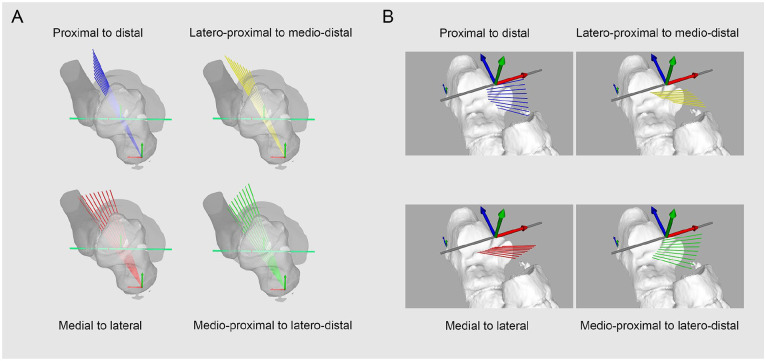
Orientation protocol of talar or calcaneal tunnel. (A) Talar and (B) calcaneal entry points located along a line following the subtalar rotation axis. Orientation of deviation planes in reference to the talocrural rotation axis. Incremental deviation of 2 degrees from 0 to 8 degrees along the talar or calcaneal entry point, either in the proximal-to-distal (blue), medial-to-distal (red), lateroproximal to medio-distal (yellow), or medioproximal to laterodistal plane (green). [See online article for color figure.]

The entry or exit point of the talar or calcaneal tunnel of the ITCL was defined as a line along the subtalar rotation axis. Incremental deviations of 2 degrees around the talar entry point and calcaneal entry point were created and allowed to simulate multiple talar and calcaneal exit points in the subtalar space. A line joining the spatial coordinates of the exit points was defined as the intraarticular portion of the ITCL by calculating their Euclidean distance in 3D space. The deviations were oriented in reference to the tibiotalar axis and oriented from proximal to distal, medial to lateral, medioproximal, to laterodistal and lateroproximal to medio-distal (Figure 2). 33 different exit points were generated on the talus and calcaneus, leading to 1089 combination of ITCL.

The length variation of all these combinations was automatically computed with a rotation in 3D space of the exit point around either the vertical rotation axis along the tibia or the subtalar rotation axis.

### Statistical Analysis

The statistical analysis was performed with IBM SPSS, version 26 (SPSS Inc, Chicago, IL). The ligament length variation at a position *α*, defined as *Length α° – Length 0°*, was calculated for each 1089 ITCL combination in 7 positions of talocalcaneal subluxation in internal and external talar rotation and 7 positions of inversion/eversion.

Further statistical analysis was restricted to 4 combinations of ITCL where the talar tunnel was at least 90% extraarticular and the calcaneal tunnel at least 70% extraarticular, which provided the highest ligament strain, expressed as positive length variation, in internal rotation and which presented the lowest strain in inversion and eversion. The distribution of length variation through the whole range was assessed with a Shapiro-Wilk test and their homogeneity of variance assessed with a Levene *F* test, both with a significance level of .05. Statistical significance was assessed with 1-way analysis of variance and post hoc analysis was performed with Games-Howell test for nonnormally distributed ITCL combination and inhomogeneous variance, and Dunnett test for normal and inhomogeneous variance.

## Results

Three outcomes were observed: (1) Extraarticular location of the tunnel in the articular surface, (2) ligament length variation through the whole range of motion of internal/external talocalcaneal subluxation and inversion/eversion, and (3) combination of tunnel for a ligament reconstruction providing the most frequent extraarticular location, having a ligament strain in internal talar rotation and isometric properties in inversion/eversion.

### Extraarticular Tunnel Location

**Figure 3.**
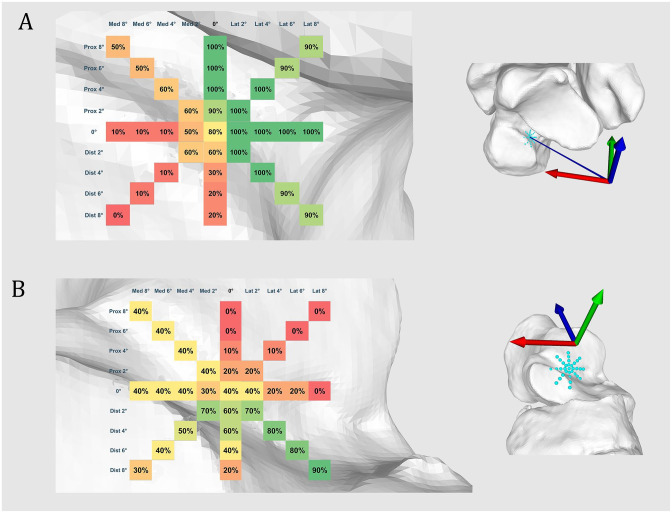
Extraarticular talar and calcaneal tunnel location. Extraarticular location of (A) talar or (B) calcaneal tunnel in the subtalar space expressed in percentage (%) of 10 ankles. From green to red: extraarticular to intraarticular intrusion. [See online article for color figure.]

Among the 10 feet, no articular intrusion of the talar tunnel was observed with proximal orientation between 4 and 8 degrees, lateral orientation between 2 and 8 degrees, lateroproximal orientation between 2 and 4 degrees, and laterodistal between 2 and 4 degrees. An extraarticular location of more than 90% could be observed with proximal orientation of 2 degrees, lateroproximal orientation between 6 and 8 degrees, and laterodistal orientation between 6 and 8 degrees (Figure 3A).

The most extraarticular locations of the calcaneal tunnel was observed with a laterodistal orientation between 2 and 6 degrees (70%) and a laterodistal orientation of 8 degrees (90%) (Figure 3B).

### Ligament Length Variation

The average length of the ITCL in neutral position is 11.3 mm (SD 3.5 mm).

**Figure 4.**
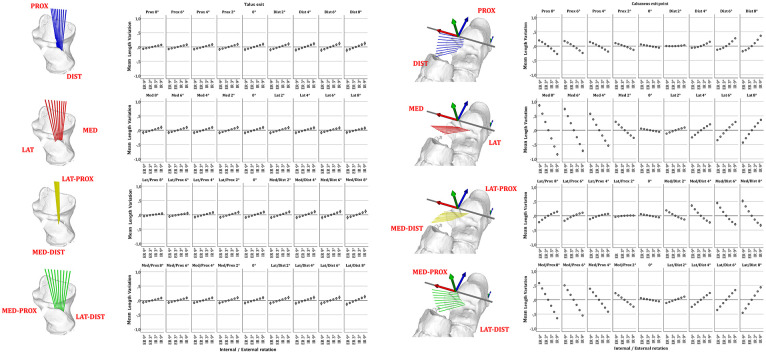
Length variation of talocalcaneal ligament according to tunnel location. Error plots depict mean ligament length variation of 10 feet from maximal internal to external peritalar rotation according to talar or calcaneal insertion point. Deviation of the talar or calcaneal tunnel around the talar or calcaneal entry point and in relation to the location of the subtalar rotation axis, with incremental deviation of 2 degrees from 0 to 8 degrees either in the proximal-to-distal (blue), medial-to-distal (red), lateroproximal to medio-distal (yellow), or medioproximal to laterodistal (green) plane. Whiskers show a CI of 95% [See online article for color figure.]

A ligament strain of the ITCL in internal talar rotation is observed with a calcaneal tunnel placement in distal, lateral, or laterodistal direction. Conversely, a proximal, medial, or medioproximal orientation of the calcaneal tunnel would produce a lengthening in external talar rotation. All the talar tunnel orientations lead to a lengthening or shortening of the ITCL respectively in internal or external talar rotation. An isolated deviation of the talar tunnel orientation has an influence on this observation only if an adequate combination of the calcaneal tunnel placement is performed (Figure 4).

### Optimal tunnel combination

**Figure 5.**
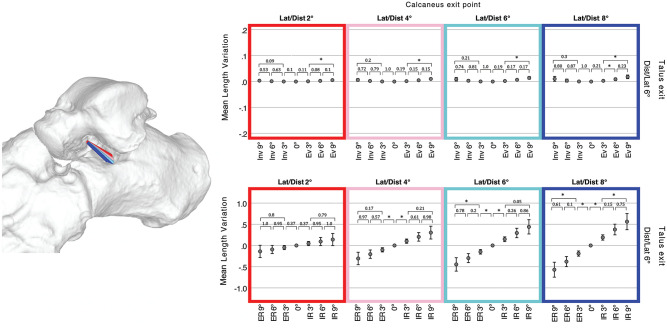
Length variation of optimal combination talocalcaneal ligaments. Four combinations of talocalcaneal ligaments having the same talar tunnel (6 degrees laterodistal oriented) and calcaneal tunnel either 2 degrees (red), 4 degrees (rose), 6 degrees (cyan), or 8 degrees (blue) laterodistal oriented. Error plots depict mean ligament length variation of 10 feet through the whole range of motion in inversion (Inv) / eversion (Ev) or internal (IR) / external talar rotation (ER). Whiskers show a CI of 95%; asterisks depict statistical significance after either Games-Howell or Dunnett post hoc test with *P* < .05 between each position. [See online article for color figure.]

Among the talar or calcaneal tunnel exits presenting, respectively, in ≥90% or ≥70% an extraarticular location, 4 combinations present the highest ligament lengthening in maximal internal talar rotation without major length variation in inversion or eversion: talar tunnel with laterodistal orientation of 6 degrees combined with calcaneal tunnel oriented laterodistal either at 2, 4, 6, or 8 degrees. A laterodistal orientation of 6 degrees of the talar tunnel with a laterodistal orientation of 8 degrees of the calcaneal tunnels presents a lengthening of +0.56 mm (SD 0.27, +3.8% of total length) in maximal internal talar rotation, and a shortening of −0.57 mm (SD 0.25 mm, −3.9%) in maximal external rotation. This combination has a near isometric in inversion/eversion, respectively: +0.01 mm (SD 0.01 mm, 0.1% of total length) to −0.01 mm (SD 0.01, 0.1% of total length) (Figure 5).

Among the 10 ankle specimens, the average 3D distance between the location of the rotation axis on the joint surface and a 6 degrees laterodistal deviated talar tunnel is 2.7 mm (SD 0.4). For an 8-degree laterodistal deviated calcaneal tunnel, this distance is 7.5 mm (SD 0.8).

A further analysis shows that a ligament lengthening in external talar rotation may also be achieved if a talar or calcaneal articular of ≤40% is tolerated. A tunnel combination talar tunnel 2 degrees distal oriented and a calcaneal tunnel 2 degrees mediodistal oriented present a ligament lengthening of +0.24 mm (SD 0.28, +4.1%) in maximal external talar rotation and shortening of −0.18 mm (SD 0.19, −2.8%) in internal rotation. This combination also presents near isometric properties in inversion/eversion, respectively: +0.02 (SD 0.02, 0.36%) to −0.02 mm (SD 0.02, 0.36%).

Independently of the articular intrusion, the tunnels providing the most important ligament lengthening in internal talar rotation would be a combination of talar tunnel 8 degrees mediodistal oriented and calcaneal tunnel 8 degrees distal oriented. This combination presents a lengthening of +0.86 mm (SD 0.22) in maximal internal talar rotation and −0.7 mm (SD 0.15) in external rotation. In maximal eversion and inversion, this combination would provide respectively a ligament lengthening of −0.5 mm (SD 0.08) and 0.47 mm (SD 0.08).

## Discussion

This kinematic simulation of the subtalar motion demonstrates that a ligament reconstruction located in the subtalar joint with limited intrusion of the articular surfaces would produce a ligament lengthening during a talocalcaneal subluxation with internal talar rotation, such as that observed in PFFD.^15^ Despite its eccentric position in relation to the subtalar rotation axis, it shows minimal length variations during inversion/eversion with near isometric variation values, which may potentially allow a physiological joint motion of the hindfoot.

Based on the available anatomical studies and our single-axis simulation model, we can hypothesize that such ligament reconstruction would correspond either to the lateral fibers of the ITCL or the CL, 2 ligaments whose lesions can be observed in similar incidences to the spring ligament in the case of PCFD.^13,22^ Confusion has long remained in the nomenclature and anatomy of ITCL, leading it to be referred either as “cruciate ligament of the tarsus,” “ligament of tarsal canal,” “oblique astragalo-calcanean ligament,” “axial ligament,” or “Hedge ligament of Farabeuf.” The ITCL and posterior part of the CL run in succession as a curtain and the ITCL may present a band-, fan-, or multiple-type morphology, typically with a 10.9 ± 2.7-mm-long talar footprint and a 9.5 ± 3.5-mm-long calcaneal footprint, both running parallel with the tarsal canal.^11^ Authors advocated, through in vivo magnetic resonance imaging analysis, that its fibers run closely along the subtalar rotation axis and the literature reports a ligament length ranging from 7 to 15 mm.^5,9,11,17,22^ A strict comparison between the available anatomical data and the results of this study, however, remains challenging as the used macroscopic landmarks are defined by their distance from the cartilage border which is a less reproducible and less precise for a mathematic simulation aiming to provide accurate ligament length variations.

The second main finding of this study is the optimal tunnel location of a ligament reconstruction that would stabilize an external talar rotation. Such stabilization would be possible if the calcaneal insertion were oriented in the opposite direction, that is, medial, proximal, or medioproximal. Noticeably, such reconstruction is achievable only if a higher probability of articular intrusion is tolerated (extraarticular location only in 70%-60% of those potentially expected for the talar or calcaneal tunnel) and the lengthening values remain lower as for a reconstruction stabilizing an internal rotation. This observation questions the use of ITCL reconstruction or CL reconstruction in cases of subtalar instability concurrent with lateral ankle instability, because such a reconstruction would be oriented in the opposite side.^10,12,18,23,30^

### Limitations

The simulation of the subtalar rotation axis may present an observer-induced variability because of the need to manually place a sphere fitting the navicular surface of the talus or calcaneal surface of the talus. To avoid such variability, 8 reference points were placed on the articular surfaces following a systematic protocol by the same observer. The cartesian coordinate of these reference points allowed to generate mathematically the spheres and the rotation axis.^19^

This study postulates that the subtalar joint motion is directly related to the bone morphology and that the subtalar axis is a rigid helical axis, which is a simplification widely used by previous in vivo studies.^9,24,27^ Simulations models have been proposed either based on generic model or on ankle models based on patient-specific talus morphology.^1,26^ Both of these models provided a helical rotation axis and have been shown to be similar, even though the subtalar rotation axis could be less accurately simulated if compared with in vivo measurement by dual-fluoroscopy. As such, a difference ranging from 0.6 to 7.6 degrees or from 1.1 to 8.9 degrees could be observed, respectively, for the talocrural and subtalar joint.^25^ This finding is consistent with an in vivo study reporting a higher variability of the helical axis of the subtalar joint compared with the talocrural joint.^28^ This can be explained by the nature of the subtalar motion which should rather be seen as a complex combination of sliding, rolling, and spinning where the ITCL and CL play a guiding important role, as they remain tensioned during the inversion and eversion.^28^ The use of a monoaxial concept in our in silico model is a useful simplification that allows to produce multiple mathematical simulations. In fact, simulating a joint motion depending on such unpredictable factors as tension of ligament with unknown biomechanical property would be highly challenging. Moreover, the aim of this study is to predict the kinematic behavior of an artificial ligament based on a simulated motion rather than to anticipate the joint behavior based on the induced ligament tension. Although biomechanically more accurate, a similar study with data obtained in vivo would either expose patients to higher levels of radiation or yield less accurate data.

As this is a strictly kinematic analysis, we cannot assert the biomechanical impact of the reported ligament lengthening. Similarly, we cannot state whether it will not alter the physiological subtalar mobility despite its near-isometric property in inversion/eversion.

The definition of the talar internal/external rotation in this study has been arbitrarily based on a vertical “subluxation axis,” which is a cylinder passing through the distal third of the tibia. The loss of joint congruence in subtalar joint, such as seen in PFFD, has been described as a medial shift of the talar head in relation to the calcaneus, which can be seen as an internal rotation.^15^ Although the loss of contact surface in the subtalar joint has been extensively quantified, the orientation of this rotational axis has not been quantified or compared to subtalar rotational axis. This vertical axis has been therefore chosen because of its reproducibility and the lack of available data reporting a subluxation axis.

The analyzed feet were retrospectively obtained through a database of reported healthy feet. No standardized weightbearing computed tomography or weightbearing radiographs have been performed to assess their alignment to exclude potentially asymptomatic flat foot or cavovarus feet, and no magnetic resonance imaging was previously performed to assess the ligaments’ conditions. However, none of the patients presented major flat foot or cavovarus deformity during clinical examination. A pathologic ankle would presumably present an atypical motion with subluxated position because of the insufficient peritalar ligaments. An in vivo kinematic analysis on pathologic feet with the same ligament simulation protocol could correct this limitation. This kinematic proof-of-concept model provides an analysis strictly in case of healthy ankles without joint incongruency, which means without insufficient ligaments, which is a key step before performing further biomechanical investigations.

The results of this study are inherently limited by its in silico design. Because it is solely based on osseous anatomy, the determination of rotation axes relies on bone rather than cartilaginous surfaces. Consequently, we presume a uniformity of cartilaginous thickness across all osseous surfaces. Likewise, impingements between the talus and calcaneus are underestimated because only bone impingement, and not cartilaginous, can be observed.

Ligament reconstructions, based on a geometric line connecting 2 points in space, do not account for any potential curvature along articular contours. A simulation of such curvature would be technically unreliable and challenging given the extensive data set proposed in this study. This limitation results in an underestimation of the ligament strain, with reconstructions exhibiting elongation due to an additional curvature.

The definition of technical feasibility is arbitrarily based on the extraarticular location of the tunnels, which is when the talar and calcaneal tunnel locations are extraarticular in, respectively, more than 90% or 70% of the specimens. This safety margin should undoubtedly be adjusted according to the thickness requirements of the reconstructed ligament.

A further biomechanical study is mandatory to determine the thickness or rigidity of an implant able to provide enough stabilizing strength. Such a subsequent study could help confirm whether a talocalcaneal ligament reconstruction can be proposed as a stand-alone procedure for early-stage PCFD, or as an intervention combined with the standard procedures such as spring ligament repair, calcaneal osteotomy, or lateral column lengthening osteotomies.

## Conclusion

This proof-of-concept study provides a better understanding of the kinematic property of different subtalar ligament reconstructions and lays the basis for biomechanical studies on surgical techniques able to address PCFD.

## Supplemental Material

sj-pdf-1-fai-10.1177_10711007231213361 – Supplemental material for Talocalcaneal Ligament Reconstruction Kinematic Simulation for Progressive Collapsing Foot Deformity Click here for additional data file. Supplemental material, sj-pdf-1-fai-10.1177_10711007231213361 for Talocalcaneal Ligament Reconstruction Kinematic Simulation for Progressive Collapsing Foot Deformity by Sylvano Mania, Silvan Beeler, Stephan Wirth and Arnd Viehöfer in Foot & Ankle International
